# Preoperative Nodal US Features for Predicting Recurrence in N1b Papillary Thyroid Carcinoma

**DOI:** 10.3390/cancers14010174

**Published:** 2021-12-30

**Authors:** Na Lae Eun, Jeong-Ah Kim, Hye Mi Gweon, Ji Hyun Youk, Eun Ju Son

**Affiliations:** Department of Radiology, Gangnam Severance Hospital, College of Medicine, Yonsei University, Seoul 06273, Korea; enrlove@yuhs.ac (N.L.E.); jakim@yuhs.ac (J.-A.K.); hyemig@yuhs.ac (H.M.G.); jhyouk@yuhs.ac (J.H.Y.)

**Keywords:** papillary thyroid carcinoma, N1b, lymph node, ultrasound, recurrence

## Abstract

**Simple Summary:**

The presence of lateral neck lymph node (LN) metastasis (N1b) is a known prognostic factor for poor prognosis and high morbidity after surgery in papillary thyroid carcinoma (PTC). Previous studies have suggested that tumor size and metastatic LN characteristics, including LN size, number, ratio, and extranodal extension, are associated with recurrence; however, the prognostic role of imaging features of LNs in the lateral neck have rarely been reported. In this study, we investigated whether ultrasound imaging features of lateral neck LN metastases can be prognostic markers for predicting recurrence and thereby guide surgical extent and posttreatment surveillance in N1b PTC.

**Abstract:**

This study aimed to investigate whether preoperative ultrasonographic (US) features of metastatic lymph nodes (LNs) are associated with tumor recurrence in patients with N1b papillary thyroid carcinoma (PTC). We enrolled 692 patients (mean age, 41.9 years; range, 6–80 years) who underwent total thyroidectomy and lateral compartment LN dissection between January 2009 and December 2015 and were followed-up for 12 months or longer. Clinicopathologic findings and US features of the index tumor and metastatic LNs in the lateral neck were reviewed. A Kaplan-Meier analysis and Cox proportion hazard model were used to analyze the recurrence-free survival rates and features associated with postoperative recurrence. Thirty-seven (5.3%) patients had developed recurrence at a median follow-up of 66.5 months. On multivariate Cox proportional hazard analysis, male sex (hazard ratio [HR], 2.277; 95% confidence interval [CI]: 1.131, 4.586; *p* = 0.021), age ≥55 years (HR, 3.216; 95% CI: 1.529, 6.766; *p* = 0.002), LN size (HR, 1.054; 95% CI: 1.024, 1.085; *p* < 0.001), and hyperechogenicity of LN (HR, 8.223; 95% CI: 1.689, 40.046; *p* = 0.009) on US were independently associated with recurrence. Preoperative US features of LNs, including size and hyperechogenicity, may be valuable for predicting recurrence in patients with N1b PTC.

## 1. Introduction

Papillary thyroid carcinoma (PTC) is the most common malignancy in the head and neck, accounting for 90% of thyroid malignancies [1,2]. PTC frequently shows lymph node (LN) metastasis in the central and lateral neck with a prevalence of approximately 20–90% [3,4,5]. In particular, the presence of LN metastasis in the lateral neck, which is staged N1b, has been associated with a high rate of posttreatment recurrence [4,6,7,8]. In addition, N1b PTC is known to be related to high morbidity rates due to extensive lateral neck dissection [9,10]. Because of its high recurrence potential and high morbidity rates, risk stratification of PTC N1b is important in patient management, including the determination of the optimal extent of surgery or posttreatment surveillance. Previous studies have reported that tumor size and metastatic LN characteristics including LN size, number, ratio, and extranodal extension are associated with the prognosis of patients with N1b PTC [6,11,12,13,14,15,16,17,18,19,20,21].

Thyroid ultrasound (US) is a noninvasive diagnostic modality that evaluates thyroid glands and LNs, and it is routinely used in the preoperative setting for staging of thyroid cancer. US features associated with LN metastasis are hyperechogenicity, cystic change, calcification, irregular or round shape, and loss of hilum [22,23,24,25]. A recent study showed that preoperative US characteristics of LNs are associated with extranodal extension in metastatic PTC [26]. Our recent study also reported that the highly suspicious features of metastatic LNs, including cystic change, calcification, or hyperechogenicity on US, were correlated with pathologic LN size, number, and extranodal extension [27]. A few studies have investigated the association between US features of the index tumor and recurrence [28,29]; however, no study has analyzed the US features of metastatic LN for predicting recurrence in patients with N1b PTC.

Therefore, we hypothesized that the specific US feature of metastatic LNs could influence recurrence-free survival (RFS) in patients with N1b PTC. Thus, the purpose of our study was to investigate whether US features of metastatic LNs are associated with recurrence in patients with N1b PTC.

## 2. Methods

### 2.1. Study Population

**Figure 1:**
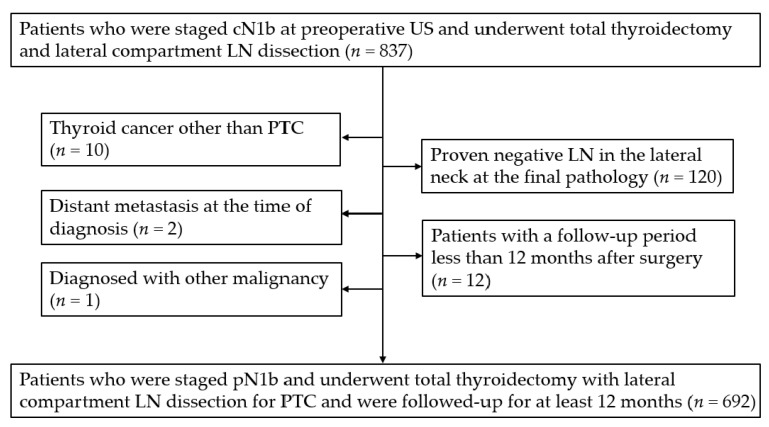
Flowchart of the study population. US, ultrasound; LN, lymph node; PTC, papillary thyroid carcinoma.

This retrospective study was approved by our Institutional Review Board and informed consent was waived. A total of 837 patients were staged N1b on preoperative US between January 2009 and December 2015 and underwent total thyroidectomy and lateral compartment LN dissection. Among them, 145 patients were excluded as follows: (a) 120 patients with proven negative LN in the lateral neck at the final diagnosis; (b) 10 patients with thyroid cancer other than PTC; (c) two patients with metastasis in other organs at the time of diagnosis; (d) one patient with other malignancy; and (e) 12 patients with a follow-up period less than 12 months after surgery. Finally, we included a total of 692 patients consisting of 464 females (mean age, 41.3 years; range, 9 to 80 years) and 228 males (median age, 43.1 years; range, 6 to 79 years) (Figure 1).

### 2.2. Image Analysis

US examinations were performed with 5–12 MHz linear array transducers (iU22; Philips Medical Systems, Best, The Netherlands), with 4–15 MHz linear array transducers (SuperSonic Imagine; Aix-en-Provence, France) and with 6–18 MHz linear array transducers (Acuson S2000; Simens Healthcare, Erlangen, Germany). Preoperative US was performed by one of six radiologists with 5 to 18 years of experience in thyroid imaging.

US images of the index cancer and metastatic LNs in the lateral neck were retrospectively reviewed by two radiologists by consensus. The tumor size was determined by the maximum diameter of the index cancer. The index cancer was assessed according to the shape (parallel and non-parallel), internal composition (solid, mixed [solid portion >50%] and cystic [solid portion <50%]), echogenicity (hyper-, iso-, hypoechoic, and marked hypoechoic), margin (smooth, lobulated, and irregular), and calcification (no calcification, macrocalcification, and microcalcification). For the lateral LNs, the index LN was determined to be the LN with suspicious features and the largest size. Imaging features of the index LN were assessed as follows: longitudinal diameter, presence of cystic change, calcification, hyperechogenicity (hyperechoic change of LN versus adjacent muscle), loss of hilum, and shape (oval, round, and irregular) [22,23,24,25].

### 2.3. Surgery and Pathologic Analysis

Total thyroidectomy with prophylactic central compartment dissection was performed. Lateral compartment LN dissection was also performed when LN metastasis was diagnosed with preoperative US-guided fine needle biopsy aspiration or intraoperative frozen section. Lateral compartment LN dissection included LNs at level II, III, IV, and anterior V.

Tumor size, multifocality, bilaterality, extrathyroidal extension, and BRAF status were recorded according to the pathologic reports. Histologic reports of lateral LNs, including the largest size, total number of harvested LNs, total number of metastatic LNs, ratio (total number of metastatic LNs/total number of harvested LNs), and extranodal extension, were also reviewed. The staging of thyroid cancer was reassessed according to the 8^th^ edition of the American Joint Committee on Cancer.

### 2.4. Postoperative Follow-Up and Recurrence

All patients underwent thyroid-stimulating hormone suppression and radioactive iodine therapy with 50–200 mCi after surgery. Based on our institution protocols, patients underwent follow-up with a neck US, a chest computed tomography (CT), and the measurement of free thyroxine, thyroglobulin (Tg), anti-Tg antibody, and serum thyroid-stimulating hormone every 12 months. Other imaging examinations, including iodine 131 whole-body scintigraphy or fluorodeoxyglucose positron emission tomography (PET)/CT, were performed in patients showing detectable serum Tg or anti-Tg antibody without evidence of recurrence on neck US or chest CT scan. No evidence of recurrence was determined with no evidence of disease on biochemistry (suppressed Tg < 1 ng/mL, stimulated Tg < 2 ng/mL, no anti-Tg antibody), on cytology, or on imaging (US, CT, iodine 131 whole-body scintigraphy, or PET/CT scan). Recurrence was defined as a newly detected disease on biochemistry, on cytology, or on imaging after a period with no evidence of disease.

### 2.5. Statistics

The disease outcome of interest was RFS. RFS was defined as the period from the date of surgery to the development of tumor recurrence in patients with recurrence or that of the most recent follow-up in patients without recurrence. The median follow-up period was 66.5 months (range, 4–127 months).

To compare demographics between the recurrence and non-recurrence groups, continuous variables were analyzed using the Student’s t-test, while categorical variables were compared using the chi-square test and Fisher’s exact test. For analyzing RFS, a univariate and multivariate Cox proportional hazard regression analysis was used to estimate hazard ratios (HRs) with 95% confidence intervals (CIs). Kaplan-Meier cumulative-event curves were constructed using the factors associated with recurrence on the multivariate Cox regression analysis and the DFS curves were compared with 95% CIs using the log-rank test. Statistical analysis was performed with SAS ver. 9.2 (SAS Institute Inc., Cary, NC, USA). *p*-values < 0.05 were considered to indicate statistical significance.

## 3. Results

Of 692 patients, 37 (5.3%) had developed recurrence. Of them, 31 patients had structural recurrence including the lateral LN (*n* = 15), the thyroid bed (*n* = 11), and five patients showed distant metastases in the lung. Six patients had biochemical recurrence showing elevated Tg levels.

Of the clinicopathologic characteristics (Table 1), the proportion of male patients was higher in the group of patients with recurrence than in the group of patients without recurrence (31.6% vs. 56.8%; *p* = 0.002). Pathologic tumor size was larger (15.7 mm vs. 20.4 mm; *p* = 0.005) and TNM stage was higher (16.9% vs. 35.1%; *p* = 0.019) in patients with recurrence. Multifocality (36.3% vs. 54.1%; *p* = 0.03), bilaterality (33.7% vs. 56.8%; *p* = 0.004), and extrathyroidal extension (80.6% vs. 94.6%; *p* = 0.034) were also more frequently observed in the recurrence group. Regarding the LN pathology, pathologic LN size (12 mm vs. 18.6 mm; *p* < 0.0001), total number of metastatic LNs (11 vs. 16; *p* < 0.0001), and LN ratio (0.23 vs. 0.3; *p* = 0.011) were greater and extranodal extension (52.5% vs. 78.4%; *p* = 0.006) was more common in patients with recurrence. Age, tumor pathology, BRAF status, and the total number of harvested LNs showed no significant difference between the two groups.

Of the US features of the index cancer (Table 2), only tumor size showed a significant difference between patients without and with tumor recurrence (17.3 mm vs. 20.8 mm; *p* = 0.044). Of the US features of the index lateral LN (Table 2), the size was larger (13.8 mm vs. 20.8 mm; *p* < 0.0001) and hyperechogenicity of LNs (61.7% vs. 94.6%; *p* < 0.0001) was more frequent in the recurrence group than in the non-recurrence group. There was no significant difference in shape, composition, echogenicity, margin, and calcification of the index tumor and cystic change, calcification, or shape of the index LN between the two groups.

According to the univariate Cox proportional hazard analysis (Table 3), male sex, age ≥55 years, tumor size and LN size at US, hyperechogenicity of LN, pathologic tumor size, multifocality and bilaterality of cancer, pathologic LN size, total number of metastatic LNs, LN ratio, and extranodal extension were significantly associated with recurrence. According to the multivariate analysis of the preoperative model (Table 4), male sex (HR, 2.321; 95% CI: 1.142, 4.360; *p* = 0.019), age ≥55 years (HR, 3.066; 95% CI: 1.537, 6.118; *p* = 0.001), larger LN size (HR, 1.055; 95% CI: 1.027, 1.084; *p* < 0.0001), and hyperechogenicity of LN (HR, 8.066; 95% CI: 1.916, 33.960; *p* = 0.004) on US were independently associated with recurrence. In the postoperative model, male sex (HR, 2.897; 95% CI: 1.332, 6.301; *p* = 0.007), larger pathologic LN size (HR, 1.065; 95% CI: 1.020, 1.111; *p* = 0.004), and higher LN ratio (HR, 27.679; 95% CI: 1.144, 669.597; *p* = 0.04) were independent factors associated with recurrence. In the combined model, male sex (HR, 2.277; 95% CI: 1.131, 4.586; *p* = 0.021), age ≥55 years (HR, 3.216; 95% CI: 1.529, 6.766; *p* = 0.002), larger LN size (HR, 1.054; 95% CI: 1.024, 1.085; *p* < 0.0001), and hyperechogenicity of LN (HR, 8.223; 95% CI: 1.689, 40.046; *p* = 0.009) on preoperative US were independently associated with recurrence.

The diagnostic performance of male sex, older age (≥55 years), LN size (20 mm), and LN hyperechogenicity for predicting recurrence are shown in Table 5. Among them, LN hyperechogenicity showed the highest area under the curve (AUC) of 0.664 with a sensitivity, specificity, positive predictive value, negative predictive value, and accuracy of 94.59%, 38.17%, 7.95%, 99.21%, and 41.18%, respectively.

**Figure 2:**
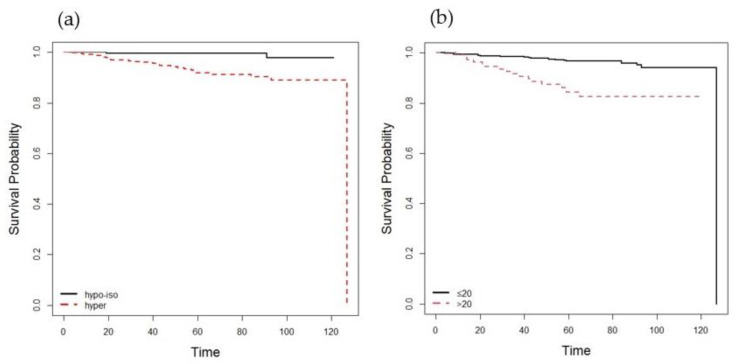
Kaplan-Meier cumulative event curves for recurrence in N1b papillary thyroid carcinoma (**a**) in patients with iso- or hypoechoic vs. hyperechoic LNs (log-rank statistic, *p* < 0.0001) and (**b**) in patients with LNs larger than 20 mm vs. LNs 20 mm or less (log-rank statistic, *p* < 0.0001). LN, lymph node.

**Figure 3:**
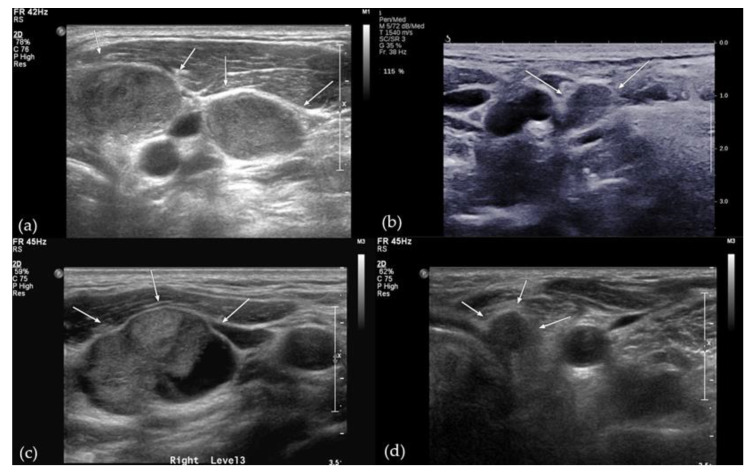
Ultrasound images showing diagnostic features of N1b papillary thyroid carcinoma. (**a**) Preoperative ultrasound image of a 24-year-old man showing hyperechoic metastatic lymph nodes on the left side of the neck at level III (arrows). (**b**) Follow-up ultrasound image taken 51 months after surgery, showing an 11-mm irregular, hyperechoic lymph node on the right side of the neck (arrows), which was a recurrence. (**c**) Preoperative ultrasound image of a 62-year-old man showing a 29-mm metastatic lymph node with hyperechogenicity and cystic change on the right side of the neck at level III (arrows). (**d**) Follow-up ultrasound image taken 48 months after surgery, showing an 8-mm irregular lymph node on the left side of the neck at level VI (arrows), which was confirmed to be a recurrence.

Patients with hyperechoic LNs showed lower 5- and 10-year RFS than patients with iso- or hypoechoic LNs (5-year RFS, 94.6% vs. 99.6%; 10-year RFS, 88.9% vs. 97.9%; both *p* < 0.0001). In addition, patients with LNs larger than 20 mm showed lower 5-year RFS than patients with LNs 20 mm or less (5-year RFS, 87.9% vs. 98.2%; 10-year RFS, 83.3% vs. 94.4%; both *p* < 0.0001) (Figure 2 and Figure 3).

## 4. Discussion

We have shown in the present study that preoperative US features of lateral LNs have the potential to predict recurrence in patients with N1b PTC. In the preoperative and combined model, US features of metastatic LNs, including the size and hyperechogenicity, were independently associated with recurrence in addition to male sex and older age (≥55 years). In our study, patients with larger (cut-off 20 mm) and hyperechoic LNs in the lateral neck had lower 5- and 10-year DFS rates than those without.

Since the metastatic LNs in the lateral neck in PTC have been associated with poorer outcomes and high morbidity rates, previous reports have investigated the clinicopathologic markers for predicting prognosis in N1b PTC. However, only a few studies have attempted to identify the imaging markers for predicting patient outcomes in N1b PTC. A previous study reported that US features of metastatic LNs including node matting, cystic area, or unclear margins were significantly associated with extranodal extension, which is an important prognostic factor in N1b PTC [26]. Similarly, our previous study showed that the suspicious US features of metastatic LNs were significantly correlated with the pathologic size, total LN number, and extranodal extension [27]. However, no studies have directly analyzed the association between US features of metastatic LNs and recurrence.

Previous studies have reported that the size of LN affected postoperative recurrence in N1b PTC [6,15,16,19,21]. A recent study showed that the presence of a metastatic LN with a maximum diameter larger than 2 cm was an independent risk factor for locoregional recurrence [15]. However, previous studies have used the pathological size of LNs. Of note, the multivariate analysis conducted in our study showed that the LN size ‘on US’ was independently associated with recurrence in the preoperative and combined model. In particular, there was a significant difference in 5- and 10-year RFS when the cut-off was 20 mm. Hyperechogenicity of the LN, which indicates the solid portion of metastatic tumors within the cortex of LNs [28], also affected RFS and showed the highest AUC value for predicting recurrence in this study. Unlike previous studies [29,30], no significant difference was found between the size and specific features of the index tumor and recurrence on the multivariate analysis. Our data suggests that the US features of metastatic LNs, including the size and hyperechogenicity, can be used for preoperative risk-stratification of N1b PTC, rather than the characteristics of the tumor itself. A previous study also suggested that the index tumor itself was not a risk factor for recurrence in N1b PTC [12].

Our results highlight that the preoperative US features of metastatic LNs can provide prognostic information ‘before’ surgery and guide the surgical management of N1b PTC. Postoperative recurrence in N1b PTC is a critical issue for both patients and surgeons due to the risk of morbidity after surgery, as well as difficulties associated with reoperation [6,10,19,31]. Considering the prognostic implication of the US features of metastatic LNs, a comprehensive LN dissection may be required to prevent recurrence, rather than selective LN removal, when the LN size is larger than 20 mm and the hyperechogenicity of LNs is identified on US. US is used as the standard mode of evaluation of PTC and has the advantages of being widely available and low cost, and there is no requirement for contrast administration. Further study is needed to validate the US features of metastatic LNs as predictors of clinical outcomes in N1b PTC.

In addition, our study showed that clinical factors such as male sex and older age were associated with recurrence, which was consistent with previous studies [6,11,12,13,15]. However, the pathologic factors, including LN size, number, ratio, and extranodal extension, which have been reported to be associated with recurrence, were not significant indicators in the multivariate analysis in this study.

We acknowledge several limitations of our study. First, this study was conducted retrospectively in a single institution and we only enrolled patients with preoperative US and pathologically confirmed lateral LNs, which may have caused selection bias. Second, the imaging assessment was performed on the largest tumor or LN, and direct node-to-node evaluation was not possible. Lastly, although the imaging features were interpreted by consensus by two radiologists, there may have been operator dependency due to the inherent nature of US.

## 5. Conclusions

Preoperative US features of LNs including the size and hyperechogenicity may be valuable for predicting recurrence in patients with N1b PTC.

## Figures and Tables

**Table 1:** Clinicopathologic characteristics of patients with N1b papillary thyroid carcinoma according to recurrence.

Characteristics	Non-Recurrence (*n* = 655)	Recurrence (*n* = 37)	*p* Value
Sex			0.002
Female	448 (68.4)	16 (43.2)	
Male	207 (31.6)	21 (56.8)	
Age (years)	41.70 ± 12.81	45.65 ± 17.58	0.186
Pathology			0.835
Conventional	545 (83.2)	30 (81.1)	
Diffuse sclerosing variant	86 (13.1)	6 (16.2)	
Others	24 (3.7)	1 (2.7)	
Pathologic tumor size (mm)	15.67 ± 9.76	20.38 ± 10.79	0.005
TNM stage			0.019
I	543 (82.9)	24 (64.9)	
II	111 (16.9)	13 (35.1)	
III	1 (0.2)	0 (0.0)	
Multifocality			0.030
No	417 (63.7)	17 (45.9)	
Yes	238 (36.3)	20 (54.1)	
Bilaterality			0.004
No	434 (66.3)	16 (43.2)	
Yes	221 (33.7)	20 (56.8)	
Extrathyroidal extension			0.034
No	127 (19.4)	2 (5.4)	
Yes	528 (80.6)	35 (94.6)	
BRAF status			0.740
No	75 (11.5)	4 (10.8)	
Yes	242 (36.9)	16 (43.2)	
None	338 (51.6)	17 (45.9)	
Pathologic LN size (mm)	11.96 ± 7.40	18.55 ± 11.24	<0.0001
Total number of harvested LNs	47.66 ± 21.15	53.91 ± 25.57	0.152
Total number of metastatic LNs	10.82 ± 8.79	16.49 ± 12.19	<0.0001
LN Ratio	0.23 ± 0.17	0.30 ± 0.14	0.011
Extranodal extension			0.006
No	38.6 (38.6)	5 (13.5)	
Yes	344 (52.5)	29 (78.4)	
None	58 (8.9)	3 (8.1)	

Values are expressed as the mean ± standard deviation or number (%). LN, lymph node; LN ratio, total number of metastatic LNs/total number of harvested LNs.

**Table 2:** Ultrasound features of the index tumor and lateral lymph nodes in patients with N1b papillary thyroid carcinoma according to recurrence.

Characteristics	Non-Recurrence (*n* = 655)	Recurrence (*n* = 37)	*p* Value
Tumor size	17.29 ± 10.37	20.84 ± 10.79	0.044
Tumor shape			0.628
Parallel	310 (47.3)	16 (43.2)	
Nonparallel	345 (52.7)	21 (56.8)	
Tumor composition			0.651
Solid	626 (95.6)	35 (94.6)	
<50% cystic	22 (3.4)	1 (2.7)	
>50% cystic	7 (1.1	1 (2.7)	
Tumor echogenicity			0.458
Hyperechogenicity	2 (0.3)	0 (0.0)	
Isoechogenicity	12 (1.8)	1 (2.7)	
Hypoechogenicity	351 (53.6)	15 (40.5)	
Markedly hypoechogenicity	290 (44.3)	21 (56.8)	
Tumor margin			0.587
Smooth	0 (0)	0 (0)	
Lobulated	102 (15.6)	7 (18.9)	
Irregular	553 (94.7)	30 (81.1)	
Tumor calcification			0.651
No	169 (25.8)	8 (21.6)	
Macrocalcification	74 (11.3)	3 (8.1)	
Microcalcification	412 (62.9)	26 (70.3)	
LN size	13.82 ± 10.37	20.84 ± 10.79	<0.0001
LN cystic change			0.345
No	472 (72.1)	24 (64.9)	
Yes	183 (27.9)	13 (35.1)	
LN calcification			0.696
No	368 (56.2)	22 (59.5)	
Yes	287 (43.8)	15 (40.5)	
LN shape			0.933
Oval	394 (60.2)	22 (59.5)	
Round/irregular	261 (39.8)	15 (40.5)	
LN echogenicity			<0.0001
Hypo-/isoechogenicity	250 (38.2)	2 (5.4)	
Hyperechogenicity	405 (61.8)	35 (94.6)	

Values are expressed as the mean ± standard deviation or number (%). LN, lymph node.

**Table 3:** Univariate Cox proportional hazard analysis for predicting recurrence in patients with N1b papillary thyroid carcinoma.

Variables	HR (95% CI)	*p* Value
Sex		
Female	reference	
Male	3.007 (1.550–5.835)	0.001
Age (years)		
<55	reference	
≥55	2.542 (1.287–5.019)	0.007
US tumor size	1.028 (1.004–1.053)	0.024
Tumor shape		
Parallel	reference	
Nonparallel	1.332 (0.686–2.586)	0.398
Tumor composition		
Not solid	reference	
Solid	0.828 (0.199–3.449)	0.796
Tumor echogenicity		
Hyper-, iso-, hypoechogenicity	reference	
Markedly hypoechogenicity	1.566 (0.811–3.021)	0.181
Tumor margin		
Smooth	reference	
Lobulated/irregular	0.000	>0.999
Tumor calcification		
No/macrocalcification	reference	
Microcalcification	1.408 (0.692–2.864)	0.345
LN size	1.070 (1.045–1.095)	<0.0001
LN cystic change		
No	reference	
Yes	1.258 (0.629–2.516)	0.516
LN calcification		
No	reference	
Yes	0.920 (0.474–1.785)	0.805
LN shape		
Oval	reference	
Round/irregular	1.093 (0.563–2.122)	0.792
LN echogenicity		
Hypo-isoechogenicity	reference	
Hyperechogenicity	10.855 (2.607–45.203)	0.001
LN hilum		
No	reference	
Yes	4.148 (0.568–30.313)	0.161
Pathologic tumor size	1.042 (1.017–1.068)	0.001
Multifocality		
No	reference	
Yes	1.979 (1.027–3.811)	0.041
Bilaterality		
No	reference	
Yes	2.364 (1.225–4.564)	0.01
Extrathyroidal extension		
No	reference	
Yes	4.144 (0.995–17.251)	0.051
BRAF status		
No	reference	
Yes	1.288 (0.430–3.855)	0.651
Pathologic LN size	1.078 (1.044–1.113)	<0.0001
Total number of harvested LNs	1.012 (1.000–1.025)	0.060
Total number of metastatic LNs	1.048 (1.023–1.074)	<0.0001
LN Ratio	2.533 (1.187–5.406)	0.016
Extranodal extension		
No	reference	
Yes	3.638 (1.402–9.438)	0.008

US, ultrasound; LN, lymph node; LN ratio, total number of metastatic LNs/total number of harvested LNs; HR, hazard ratio; CI, confidence interval.

**Table 4:** Multivariate Cox proportional hazard analysis for predicting recurrence in patients with N1b papillary thyroid carcinoma.

Variables	Preop Model		Postop Model		Combined Model	
	HR (95% CI)	*p* Value	HR (95% CI)	*p* Value	HR (95% CI)	*p* Value
Male sex	2.231 (1.142–4.360)	0.019	2.897 (1.332–6.301)	0.007	2.277 (1.131–4.586)	0.021
Age ≥ 55 years	3.066 (1.537–6.118)	0.001	1.935 (0.822–4.557)	0.131	3.216 (1.529–6.766)	0.002
US features						
Tumor size	1.017 (0.993–1.042)	0.164			0.983 (0.937–1.032)	0.494
LN size	1.055 (1.027–1.027)	<0.0001		–	1.054 (1.024–1.085)	<0.0001
LN hyperechogenicity	8.066 (1.916–33.960)	0.004			8.223 (1.689–40.046)	0.009
Pathologic features						
Tumor size			1.012 (0.981–1.044)	0.460		
Multifocality			1.238 (0.525–2.919)	0.625	1.011 (0.467–2.191)	0.977
Bilaterality			1.560 (0.676–3.600)	0.298	1.838 (0.869–3.887)	0.111
LN size			1.065 (1.020–1.111)	0.004		
Total number of metastatic LNs			1.008 (0.964–1.055)	0.722	1.028 (0.988–1.069)	0.170
LN ratio			27.679 (1.144–669.597)	0.041	2.604 (0.543–12.480)	0.231
Extranodal extension			1.813 (0.668–4.923)	0.243	1.764 (0.665–4.685)	0.254

US, ultrasound; LN, lymph node; LN ratio, total number of metastatic LNs/total number of harvested LNs; HR, hazard ratio; CI, confidence interval.

**Table 5:** Diagnostic performance of clinical and ultrasound imaging features for predicting recurrence in patients with N1b papillary thyroid carcinoma.

Variables	Sensitivity (%)	Specificity (%)	PPV (%)	NPV (%)	Accuracy (%)	AUC
Male sex	56.76 (40.80–72.72)	68.40 (64.84–71.96)	9.21 (5.46–12.96)	96.55 (94.89–98.21)	67.77 (64.29–71.25)	0.626 (0.543–0.709)
Age ≥ 55 years	35.14 (19.76–50.52)	82.90 (80.02–85.78)	10.40 (5.05–15.75)	95.77 (94.11–97.43)	80.35 (77.39–83.31)	0.590 (0.511–0.669)
LN size	43.24 (27.28–59.20)	85.65 (82.97–88.33)	14.55 (7.96–21.14)	96.39 (94.87–97.91)	83.38 (80.61–86.15)	0.644 (0.562–0.726)
LN hyperechogenicity	94.59 (87.30–100)	38.17 (34.45–41.89)	7.95 (5.42–10.48)	99.21 (98.12–100)	41.18 (37.51–44.85)	0.664 (0.622–0.705)

AUC, the area under the receiver operating characteristic curve; LN, lymph node; NPV, negative predictive value; PPV, positive predictive value. The values in parentheses are 95% confidence intervals.

## Data Availability

The data presented in this study are available on request from the corresponding author. The data are not publicly available due to confidentiality reasons.
